# Clove oil and selenium nanoparticles as eco-friendly antifungal agents for photographic preservation

**DOI:** 10.1007/s00253-026-13730-3

**Published:** 2026-02-21

**Authors:** Heba El-Sayed, Mohamed E. Osman, Nesma Ali, Eslam T. Mohamed

**Affiliations:** 1 Botany and Microbiology Department, Faculty of Science, Helwan University, Helwan, 11795 Egypt; 2 Ministry of Tourism and Antiquities, Cairo, Egypt

**Keywords:** Silver gelatin photographs, *Aspergillus flavus*, Essential oils, Selenium nanoparticles, Eco-friendly antifungal agents, Molecular docking

## Abstract

**Abstract:**

Microbial biodeterioration represents a major challenge in the conservation of photographic heritage, particularly silver gelatin prints. In this study, the antifungal efficacy of clove essential oil (*Syzygium aromaticum*) and selenium nanoparticles (SeNPs) was evaluated separately against *Aspergillus flavus*. Both treatments significantly inhibited fungal growth and sporulation, with SeNPs showing superior activity at lower concentrations, while clove oil exhibited strong inhibition at higher doses. Computational analyses revealed distinct mechanisms: clove oil phytochemicals targeted ergosterol biosynthesis, cell wall organization, and lipid metabolism, whereas SeNPs induced oxidative stress and disrupted antioxidant defenses. This work provides the first integrated experimental and computational framework applying these eco-friendly agents directly to photographic materials, establishing a mechanistic basis for sustainable antifungal strategies in heritage preservation.

**Key points:**

• *Clove oil and selenium nanoparticles effectively prevent fungal damage to photographs.*

• *Different antifungal mechanisms were observed through computational analyses.*

• *Provides a sustainable, eco-friendly strategy for cultural heritage preservation.*

**Supplementary information:**

The online version contains supplementary material available at 10.1007/s00253-026-13730-3.

## Introduction

The preservation of cultural heritage requires not only control of environmental conditions but also targeted strategies against biological agents that accelerate deterioration. Silver gelatin photographs, which dominated photographic practice throughout the twentieth century, remain among the most vulnerable objects in archives and private collections. Their composite structure, consisting of a paper support, a baryta layer, and a gelatin-silver emulsion, makes them highly sensitive to both chemical degradation and microbial colonization. Gelatin and paper are hygroscopic and nutrient-rich, creating favorable conditions for microbial growth when exposed to high humidity (Ali 2020).

Chemical and microbial mechanisms often act synergistically in the deterioration of silver gelatin prints. Environmental fluctuations such as elevated relative humidity and temperature accelerate the oxidation of metallic silver into ionic forms, which may redeposit as silver sulfide, leading to image fading and yellow-brown discoloration. Simultaneously, fungi secrete hydrolytic enzymes and organic acids that weaken the gelatin binder, while pigmented metabolites cause irreversible staining (Hamza 2015). This combined action highlights the importance of antifungal strategies that not only prevent microbial growth but also reduce fungus-mediated chemical degradation of the photographic substrate.

Among the biodeteriogens affecting archival materials, *Aspergillus flavus* is particularly destructive because of its enzymatic capacity and adaptability to a wide range of environmental conditions. It thrives in relative humidity levels above 80% and moderate to warm temperatures, which are common in poorly controlled storage environments. Its ability to colonize gelatin-rich emulsions results in structural weakening, binder loss, and eventual image detachment. This destructive potential has been documented in studies on fungal contamination of heritage collections and emphasizes the need for targeted preventive measures (Gadd et al. 2024). Other fungi frequently associated with photographic biodeterioration include *Aspergillus niger*, *Aspergillus sojae*, *Penicillium oxalicum*, *Penicillium chrysogenum*, and *Epicoccum nigrum*, which contribute to substrate degradation through hydrolytic enzyme production and pigmented staining that weakens cellulose and gelatin binders (Portugal 2016; Fouda et al. 2019).

Recent conservation research has increasingly prioritized eco-friendly antifungal alternatives to mitigate the toxicity risks and substrate damage associated with conventional chemical fungicides. In this search for sustainable strategies, clove essential oil (*Syzygium aromaticum*) has emerged as a leading natural candidate. While its strong bioactivity against *Aspergillus* species is well-documented and largely attributed to eugenol’s disruption of fungal cell membranes and metabolic processes (Zahraoui 2025), its environmental profile is equally critical. Clove oil is widely regarded as a safe alternative; it is classified as generally recognized as safe (GRAS) by the U.S. Food and Drug Administration (FDA) and exhibits high biodegradability, ensuring it does not accumulate as a persistent environmental pollutant (Fathiraja et al. 2024; Shende and Shilpashree 2024). In parallel, selenium nanoparticles (SeNPs) have emerged as advanced antimicrobial agents, acting through oxidative stress induction and interference with cellular redox balance (Serov et al. 2023). Furthermore, studies have demonstrated that SeNPs exhibit significantly lower toxicity and higher biocompatibility compared to inorganic selenium salts while maintaining potent antimicrobial efficacy (Mikhailova 2023; Das et al. 2025). This study aims to evaluate the antifungal potential of clove essential oil and selenium nanoparticles against *A. flavus*, the primary fungal threat to silver gelatin photographs. The antifungal efficacy of both strategies has been well-documented in previous pharmaceutical and food safety research. The bioactivity of clove essential oil is supported by recent studies demonstrating its membrane-disrupting properties (Tian et al. 2022; Allizond et al. 2023; Liñán-Atero et al. 2024), while the antimicrobial potential of SeNPs has been validated for their ability to induce oxidative stress (Shakibaie et al. 2015; Serov et al. 2023; Shahbaz et al. 2023). However, this study establishes novelty by applying these agents directly to photographic gelatin binders, a unique matrix distinct from food or clinical substrates, and integrating computational insights to map their mechanisms.

## Materials and methods

### Sampling and isolation of fungal contaminants from historical silver gelatin prints

Four silver gelatin photographs, designated a, b, c, and d (Supplementary Fig. S1), dating from the early twentieth century, exhibited visible biological deterioration. These samples were obtained from the Dr. Francis Amin Private Collection specifically for this study, and permission for their analysis and scientific publication was granted by the owner. The photographs had been stored in suboptimal environmental conditions, characterized by elevated humidity (> 65%) and fluctuating temperatures. Furthermore, the prints were stored in direct physical contact without protective interleaving. This stacking created humid microclimates between the gelatin layers and facilitated cross-contamination of fungal spores from infected to adjacent clean surfaces, thereby significantly enhancing the risk of biodeterioration. Samples were collected aseptically and transported to the Mycology Laboratory in the Faculty of Science at Helwan University, Egypt, for microbiological investigation.

Fungal isolation was achieved by swabbing the visibly deteriorated areas of the photos with sterile cotton swabs, followed by streaking on potato dextrose agar (PDA) plates. The plates were incubated at 28 °C for 7 days. Resulting fungal colonies were enumerated and identified based on macroscopic cultural features (including colony color, texture, reverse pigmentation, and growth rate) as well as microscopic morphological characters (such as conidiophore structure and spore ornamentation), applying the taxonomic keys of Gilman (1957) and Moubasher (1993). For quantitative evaluation of species distribution, frequency of occurrence (Fq) and relative density (Rd) were calculated using the following formulas:

$$Fq=\frac{Np}{Nt}\times 100$$

$$Rd=\frac{ni}{Ntotal}\times 100$$

where *Np* is the number of samples containing the species, *Nt* is the total number of samples, *ni* is the number of isolates of the individual species, and *Ntotal* is the total number of all fungal isolates obtained (Jedidi et al. 2018).

### Molecular identification of the most common isolated fungal species

The dominant fungal isolate from the deteriorated silver gelatin photographs was molecularly identified to confirm its taxonomy. For this, it was first cultured on PDA and incubated at 28 °C for 7 days, then mycelial biomass was harvested for DNA extraction using the GeneJET Genomic DNA Purification Kit (Thermo Scientific, Waltham, MA, USA), following the manufacturer’s protocol as described by Abdel-Maksoud et al. (2022). The internal transcribed spacer (ITS) region was amplified using universal primers ITS1 and ITS4, under standard PCR conditions: initial denaturation at 94 °C for 3 min, followed by 30 cycles of 94 °C for 30 s, 55 °C for 30 s, and 72 °C for 1 min, with a 10 min final extension at 72 °C (Abdel-Maksoud et al. 2022). Amplicon verification was performed in a 1.5% agarose gel electrophoresis stained with ethidium bromide. Purified PCR products were sequenced bidirectionally by Colors Lab (Cairo, Egypt) and compared against GenBank (https://www.ncbi.nlm.nih.gov/genbank/) using BLASTn (https://blast.ncbi.nlm.nih.gov/). A sequence identity of ≥ 99% with reference strains was considered conclusive for species-level identification.

### Antifungal activity of essential oils and selenium nanoparticles against *A. flavus*

Four essential oils—clove (*S. aromaticum*), garlic (*Allium sativum*), lavender (*Lavandula* sp.), and tea tree (*Melaleuca alternifolia*)—were purchased from the Natural Oils Department, National Research Center (Cairo, Egypt), and stored at 4 °C in dark bottles until use. Commercial selenium nanoparticles (SeNPs, 80 nm particle size, 0.15 wt.% aqueous suspension, Catalog No. 919519) were obtained from Sigma-Aldrich (Taufkirchen, Germany).

Antifungal screening was carried out by incorporating each essential oil or SeNPs into molten PDA (45–50 °C) at a final concentration of 5 µL/mL. The medium was thoroughly mixed, poured into sterile 9-cm Petri dishes, and allowed to solidify. Plates without additives served as controls. Each plate was centrally inoculated with a 5-mm disc taken from the margin of a 7-day-old culture of *A. flavus* (AUMC15234; GenBank: MZ945517) and incubated at 25 °C for 6 days. Colony diameters were measured along two perpendicular axes, and inhibition rate (IR%) was calculated as follows:

$$\mathrm{IR}\%=\frac{\mathrm{R}1-\mathrm{R}2}{\mathrm{R}1}\times 100$$

where R1 is radial growth in the control and R2 is radial growth in treatment (Naglah et al. 2021).

For broth assays, graded concentrations of clove oil (3–14 µL/mL) and SeNPs (0.2–1.0 µL/mL) were incorporated into 100 mL PDB in 250-mL conical flasks before inoculation with 5-mm mycelial plugs. Flasks were incubated at 25 °C for 7 days. Mycelial mats were harvested, washed, and dried at 60 °C to constant weight. The inhibition efficiency (IE%) was calculated as follows:

$$\mathrm{IE}\%=\frac{W\mathrm{c}-W\mathrm{t}}{W\mathrm{c}}\times 100$$

where *W*c is the dry weight of the control and *W*t is that of the treated samples (Geweely et al. 2019). Sporulation was recorded microscopically and macroscopically across all treatments.

### Gas chromatography-mass spectrometry (GC–MS) of clove essential oil

Clove essential oil was analyzed by GC–MS to determine volatile composition and relative abundance. An aliquot (1 µL) of the pure oil was injected (split mode, split ratio 1:50) into an Agilent/HP-type GC–MS system (Agilent Technologies, Santa Clara, CA, USA) equipped with a non-polar capillary column (e.g., HP-5MS, 30 m × 0.25 mm i.d., 0.25 µm film). The injector and MS transfer line were maintained at ~ 250–280 °C, and ionization was achieved by electron impact at 70 eV. The oven temperature program began at 50 °C (hold 2 min), ramped at 3–5 °C/min to 150 °C, then 10 °C/min to 280 °C (final hold 5–10 min); helium was used as carrier gas at ~ 1.0 mL/min. Compound identification combined mass spectral matching against commercial libraries (NIST/Wiley, Hoboken, NJ, USA) and comparison of experimental linear retention indices (calculated relative to *n*-alkane standards) with literature values and authenticated standards, when available. This identification strategy follows established practice for essential-oil analysis and GC–MS reporting (Gamal El-Din et al. 2022).

### Application of clove essential oil and selenium nanoparticles on photographic preservation

To serve as standardized model substrates for antifungal testing, new black-and-white photographs were prepared using neutral-black, multigrade fiber-based silver gelatin paper with high light sensitivity (Alsora Almoa’asera Studio, Cairo, Egypt). These were printed by a traditional technique involving exposure of glass negatives to light, development with hydroquinone (Foma developer; Foma Bohemia, Hradec Králové, Czech Republic), fixation with sodium thiosulfate (Foma fixer), and thorough washing, then sterilized under UV light (254 nm, 30 min per side) in a laminar flow cabinet to eliminate surface contaminants. Biodeterioration was simulated using *A. flavus* maintained on PDA at 28 °C; spore suspensions were prepared by flooding 7-day-old cultures with 10 mL sterile saline solution (8.5 g NaCl/L H₂O) containing 0.01% Tween 80, scraping with a sterile loop, filtering through sterile gauze to remove hyphae, and adjusting to 1 × 10⁶ spores/mL using a hemocytometer. Sterile photographic samples (2 × 2 cm) were inoculated with 100 µL of spore suspension and incubated at 28 °C under 80–85% relative humidity in sealed Petri dishes lined with moist filter paper, with each treatment performed in triplicate, while negative controls included sterile uninoculated samples and positive controls included inoculated untreated samples. Antifungal treatments consisted of clove essential oil prepared as an emulsion in 1% Tween 80 at 14 µL/mL and selenium nanoparticles (SeNPs) at 1.5 µg/mL; both were applied separately by spraying 200 µL on each side of the photographs while avoiding disturbance of the gelatin layer. Preventive assays were also conducted by applying treatments to sterilized photographs before inoculation with *A. flavus*. All treated samples were incubated under the same conditions and examined daily for 10 days; at the end of the incubation, swabs were taken aseptically from treated surfaces and inoculated onto PDA, with plates incubated at 25 °C for 7 days to detect microbial growth. The effectiveness of treatments was assessed through the absence or reduction of visible fungal growth, lack of microbial colonies on culture media, and preservation of the gelatin binder compared with untreated controls (Cinteză and Tănase 2020; Corbu et al. 2023; Menicucci et al. 2023).

### Computational insights into the antifungal mechanisms of clove essential oil constituents and selenium nanoparticles against *A. flavus*

A dual computational strategy was applied to elucidate the antifungal mechanisms of clove essential oil constituents and selenium nanoparticles against *A. flavus*. The phytochemicals identified by GC–MS, including eugenol, β-caryophyllene, eugenyl acetate, caryophyllene oxide, and fatty acids, were analyzed using the SwissTargetPrediction web server (http://www.swisstargetprediction.ch/) to identify their most probable protein targets. Since the database is largely built on mammalian proteins, the predicted targets were mapped to fungal orthologs by conducting BLASTp searches (https://blast.ncbi.nlm.nih.gov/) against the *A. flavus* proteome and cross-referencing with UniProt (https://www.uniprot.org/). These validated fungal targets were then positioned within metabolic and signaling pathways using KEGG (https://www.kegg.jp/) and Reactome (https://reactome.org/) databases to establish mechanistic relevance. In parallel, the antifungal potential of selenium nanoparticles (SeNPs) was modeled on the basis of their physicochemical reactivity. Unlike small-molecule inhibitors, nanoparticles act predominantly through redox perturbation and induction of oxidative stress. Thus, KEGG pathway mapping was used to identify antioxidant defense systems, including glutathione metabolism, peroxisomal reactive oxygen species (ROS) detoxification, and mitogen-activated protein kinase (MAPK) stress-signaling, as primary sites of SeNP-mediated interference (Shahbaz et al. 2023).

### Network pharmacology and protein–protein interaction analysis of clove oil constituents and selenium nanoparticles

Clove oil constituents and SeNPs were investigated using a pharmacology network approach to reveal their systemic antifungal mechanisms against *A. flavus*. Predicted targets were integrated into protein–protein interaction (PPI) networks using STRING v12.0 (https://string-db.org/) with *Saccharomyces cerevisiae* orthologs as a model system for fungal pathways. For clove oil, KEGG pathway enrichment highlighted functional modules associated with sterol biosynthesis, cell wall organization, and lipid metabolism. SeNP activity was evaluated by assembling oxidative stress–related proteins, including superoxide dismutase (SOD), catalase (CAT), glutathione peroxidase (GPx), and thioredoxin reductase (TrxR) into a STRING-based PPI network.

### Molecular docking analysis of phytochemicals and selenium nanoparticles with *A. flavus* proteins

Molecular docking simulations were employed to evaluate the interactions of major clove essential oil constituents (eugenol, caryophyllene oxide, and eugenyl acetate) and selenium nanoparticles (SeNPs) with key *A. flavus* proteins associated with ergosterol biosynthesis, cell wall integrity, lipid metabolism, and oxidative stress responses. Protein structures were prepared through homology modeling using the SWISS-MODEL platform (https://swissmodel.expasy.org/), referencing the UniProt sequences of sterol 14-α demethylase CYP51A (B8N2C8), chitin synthase (A0A364MBE7), HMG-CoA reductase (A0A5N6H1K4), and fatty acid synthase β (B8NL80). For the oxidative stress network targeted by SeNPs, models of superoxide dismutase (Q8X1S6), catalase (A0A5N6GI65), glutathione peroxidase-like protein (A0A5N6H0W2), and thioredoxin reductase (A0A364M3B4) were used. Ligands were energy-minimized using OPLS3e force fields, and docking was performed with Maestro 11.5 (Schrödinger, LLC, New York, NY, USA). Docking scores were calculated to predict binding affinities (kcal/mol), and hydrogen bond interactions were analyzed to identify critical protein–ligand contacts.

### Statistical analysis

All experiments were carried out in triplicate, and results are presented as mean ± standard deviation (SD). Data were analyzed using one-way ANOVA followed by Tukey’s post hoc test, with *p* < 0.05 considered statistically significant. Statistical analyses were performed using SPSS v28 (IBM Corp., Armonk, NY, USA).

## Results

### Isolation of fungal contaminants from historical silver gelatin prints

Six fungal species were isolated from the degraded silver gelatin photographs. *A. flavus* showed the highest prevalence, with a relative density of 38.5% and an occurrence frequency of 100%. *A. sojae* and *P. oxalicum* followed, each with 15.38% relative density and 50% frequency. *A. niger*, *P. chrysogenum*, and *E. nigrum* were less common, each exhibiting 7.69% relative density and 25% frequency. The consistent dominance of *A. flavus* across all examined photographs highlights its role as the primary biodeteriogen in this collection (Supplementary Fig. S2).

### Molecular identification of the most frequent deteriorated fungus

ITS region sequencing of the dominant fungal isolate produced a clear amplicon of approximately 600 bp. BLASTn analysis revealed 100% identity with *A. flavus* reference sequences deposited in GenBank. The consensus sequence generated from this study was submitted to GenBank and assigned the accession number MZ945517. The identified fungus was deposited as *A. flavus* AUMC15234 at Assiut University, Mycological Center, Faculty of Science, Assiut, Egypt. Molecular identification therefore confirmed *A. flavus* as the principal biodeteriogen associated with the degraded silver gelatin prints, fully supporting the morphological identification (Supplementary Fig. S3).

### Antifungal activity of essential oils and selenium nanoparticles against *A. flavus*

**Fig. 1:**
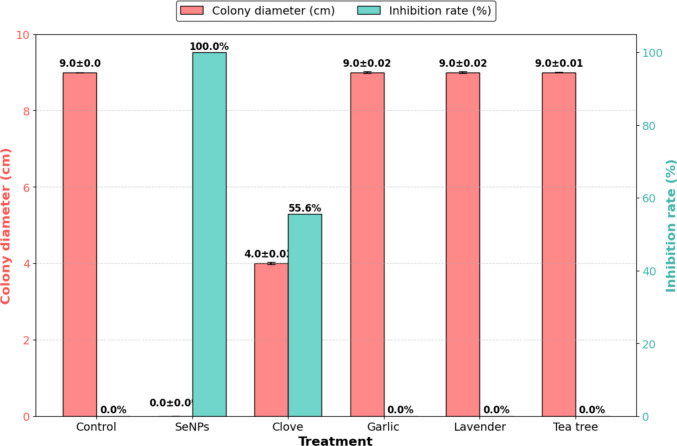
Comparison of colony diameter and inhibition rate (%) of *A. flavus* under different treatments. Data are presented as mean ± SD (*n* = 3). Asterisks indicate significant differences compared to the control (*p < 0.05; **p < 0.01; ***p < 0.001)

Significant variation in antifungal efficacy was observed among the tested treatments (Fig. 1). Selenium nanoparticles (SeNPs) completely suppressed the growth of *A. flavus* at 5 µL/mL, resulting in 100% inhibition and the absence of visible colony development. Clove essential oil markedly reduced colony diameter to 4.0 ± 0.03 cm, corresponding to 55.6% inhibition. Conversely, garlic, lavender, and tea tree oils did not exhibit measurable inhibitory activity at the tested concentration, with colony diameters comparable to the untreated control (9.0 cm).

**Fig. 2:**
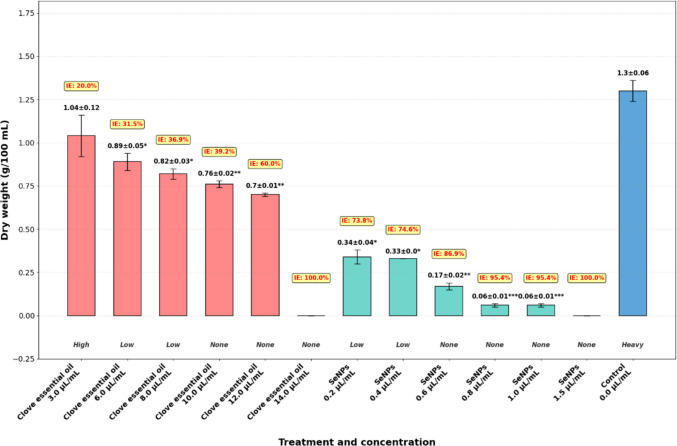
Dry weight and inhibition efficiency of *A. flavus* after treatment with essential oils and SeNPs. Data are presented as mean ± SD (*n* = 3). Conidia formation categories (heavy, high, low, none) are shown below bars. Asterisks indicate significant differences compared to control (*p < 0.05; ** *p* < 0.01; *** *p* < 0.001)

Broth assays (Fig. 2) revealed concentration-dependent effects of clove oil and SeNPs on fungal biomass and sporulation. Clove oil progressively decreased the dry weight of *A. flavus* mycelia, with inhibition efficiency (IE%) increasing from 20% at 3 µL/mL to complete suppression (100%) at 14 µL/mL. Sporulation was substantially reduced at concentrations ≥ 6 µL/mL and was completely absent at ≥ 10 µL/mL. SeNPs exhibited higher potency at lower concentrations, reducing fungal biomass by > 70% at 0.2 µL/mL and achieving near-complete inhibition (> 95%) between 0.8 and 1.0 µL/mL. Sporulation was inhibited at ≥ 0.6 µL/mL, and no viable conidia were detected at higher concentrations. In contrast, control flasks supported vigorous mycelial growth with a mean dry weight of ~ 1.3 ± 0.06 g/100 mL and abundant sporulation. Collectively, these results demonstrate that clove oil and SeNPs were the only treatments with pronounced antifungal activity, with SeNPs displaying superior efficacy.

### Gas chromatography-mass spectrometry (GC–MS) of clove essential oil

**Fig. 3:**
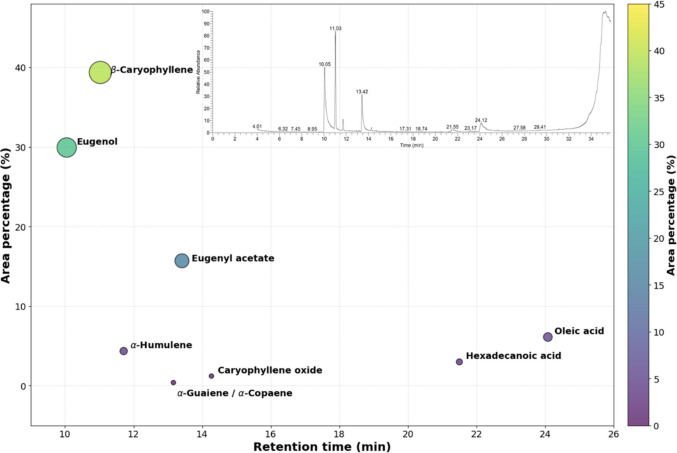
GC–MS chromatogram of clove essential oil components

GC–MS profiling of the clove essential oil produced a chromatogram in which the components were identified and quantified by percentage area. β-Caryophyllene was the most abundant constituent at 39.35% (retention time (RT) 11.03 min), followed by eugenol (2-methoxy-4-(2-propenyl)phenol) at 29.92% (RT 10.05 min) and eugenyl acetate at 15.69% (RT 13.41 min). Other detected components included oleic acid (6.11%, RT 24.08 min), α-humulene (4.35%, RT 11.71 min), and hexadecanoic acid (2.99%, RT 21.50 min). Minor constituents included caryophyllene oxide (1.21%, RT 14.27 min) and α-guaiene/α-copaene (0.39%, RT 13.16 min). These percentages were reported as GC peak-area percent of total ion current (Fig. 3).

### The application of clove essential oil and selenium nanoparticles on photographic preservation

**Fig. 4:**
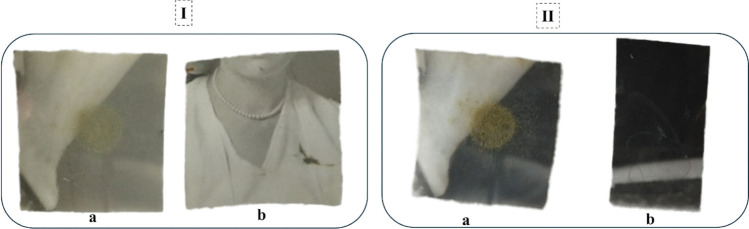
Representative photographs of treated and infected samples. **I** Experimental treatment showing (a) photograph inoculated with *A. flavus* and (b) photograph treated with clove essential oil. **II** Experimental treatment showing (a) a photograph inoculated with *A. flavus* and (b) a photograph treated with selenium nanoparticles

Untreated photographic samples inoculated with *A. flavus* exhibited rapid colonization characterized by dense hyphal penetration into the gelatin, baryta, and paper layers, accompanied by visible staining and emulsion deterioration. Samples treated with clove essential oil showed complete suppression of fungal growth; no hyphae were observed visually, and swab cultures on PDA media remained sterile throughout the 10-day incubation. SeNP- treated samples displayed substantial fungal inhibition, with mycelia appearing thin, fragmented, and morphologically compromised, although occasional fungal traces were noted in comparison to untreated controls. Negative controls (sterile, non-inoculated samples) remained clear, and positive controls (inoculated, untreated samples) developed severe fungal growth, confirming assay validity. Treatment integrity was verified by the absence of mechanical disturbance or visible alteration to the gelatin layer in all treated photographs (Fig. 4).

### Computational insights into the antifungal mechanisms of clove essential oil constituents and selenium nanoparticles against *A. flavus*

**Fig. 5:**
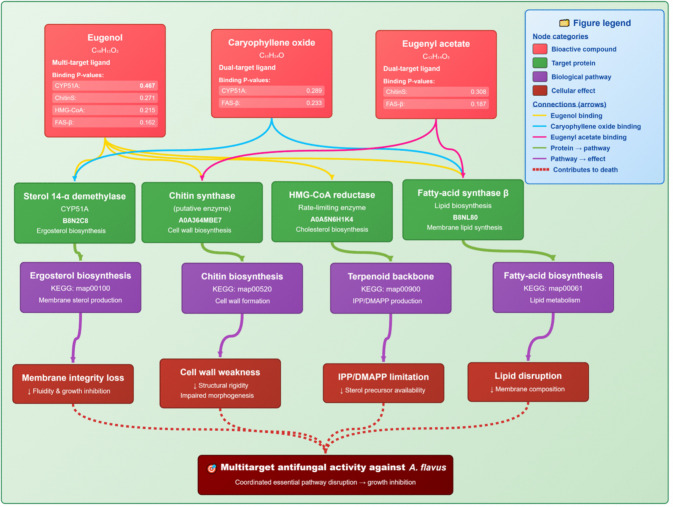
Predicted molecular targets and KEGG pathway mapping of clove oil constituents (eugenol, caryophyllene oxide, and eugenyl acetate) against *A. flavus*, highlighting multi-target disruption of ergosterol, chitin, terpenoid, and lipid biosynthetic processes

Computational analysis of clove oil constituents and selenium nanoparticles against *A. flavus* revealed distinct yet complementary antifungal mechanisms. The three major phytochemicals including eugenol, caryophyllene oxide, and eugenyl acetate were predicted to interact with multiple fungal enzymes and metabolic pathways essential for growth and survival. Eugenol demonstrated multi-target potential, with significant predicted interactions against sterol 14-α demethylase (CYP51A), chitin synthase, HMG-CoA reductase, and fatty-acid synthase, implicating ergosterol biosynthesis, cell wall formation, terpenoid backbone biosynthesis, and lipid metabolism as primary sites of interference. Caryophyllene oxide acted as a dual-target ligand, influencing both CYP51A and fatty-acid synthase, suggesting an ability to simultaneously disrupt membrane sterol production and cell-wall rigidity. Eugenyl acetate showed dual-target behavior as well, with predicted inhibition of chitin synthase and fatty-acid synthase, which may weaken morphogenesis and membrane lipid balance. Together, these compounds appear to impair fungal physiology through a coordinated attack on ergosterol biosynthesis, chitin-dependent wall integrity, terpenoid precursor availability, and lipid homeostasis. The convergence of these pathways explains the broad-spectrum antifungal activity of clove oil, with eugenol serving as the dominant bioactive supported by caryophyllene oxide and eugenyl acetate (Fig. 5).

**Fig. 6:**
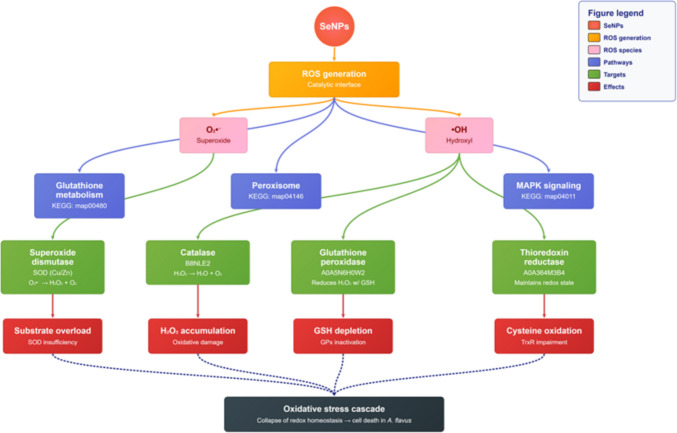
Proposed antifungal mechanism of selenium nanoparticles (SeNPs) against *A. flavus*, showing ROS generation, perturbation of antioxidant defense pathways, and progression toward oxidative stress–induced cell death

In contrast, SeNPs exhibited a fundamentally different mode of action centered on redox disruption. Their catalytic surface properties promoted continuous generation of ROS, including superoxide anions (O₂^•⁻^) and hydroxyl radicals (^•^OH), which overwhelmed the fungal antioxidant defense system. Key detoxification enzymes such as SOD, CAT, and glutathione peroxidase (GPx-like) were predicted to become overloaded or inactivated, leading to accumulation of H₂O₂, depletion of glutathione, and lipid peroxidation. Moreover, impairment of thioredoxin reductase was associated with the collapse of redox homeostasis and dysregulation of MAPK stress signaling. The combined effect of these disruptions is an oxidative stress cascade resulting in lipid, protein, and DNA damage, ultimately culminating in fungal cell death. Taken together, the results highlight that clove oil constituents predominantly act through inhibition of structural biosynthetic pathways, while SeNPs disrupt antioxidant defenses through ROS overproduction. The complementary nature of these mechanisms suggests potential for synergistic antifungal applications in managing *A. flavus* (Fig. 6).

### Network pharmacology and protein–protein interaction analysis of clove oil constituents and selenium nanoparticles

**Fig. 7:**
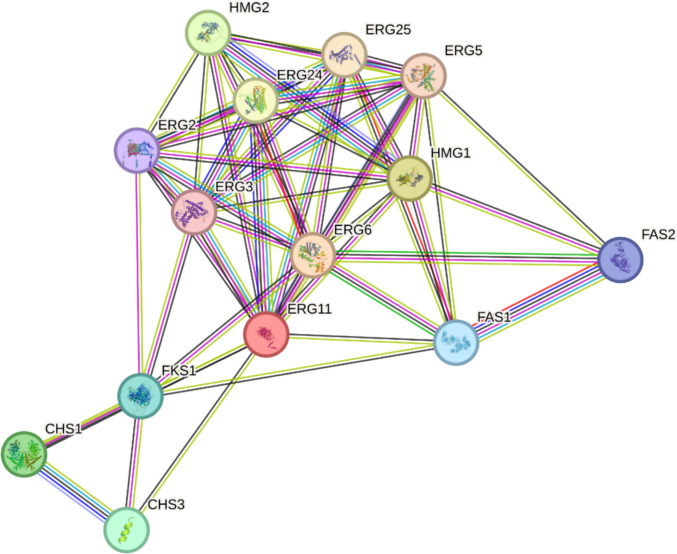
Protein–protein interaction (PPI) network of predicted fungal targets of clove oil constituents, highlighting sterol biosynthesis (ERG cluster), cell wall organization (CHS, FKS1), and lipid metabolism (FAS1/2) as interconnected antifungal modules

The PPI interactome of clove oil constituents resolved into three interlinked functional modules: sterol biosynthesis, cell wall assembly, and fatty acid metabolism. The ergosterol module formed the central hub, anchored by lanosterol 14α-demethylase (ERG11), sterol *C*-methyltransferase (ERG6), and HMG-CoA reductases (HMG1/2). Multiple clove constituents, including eugenol, eugenyl acetate, and caryophyllene oxide, converged on this pathway, indicating a multi-target blockade of ergosterol biosynthesis. A secondary cluster was defined by chitin synthases (CHS1/CHS3) and β−1,3-glucan synthase (FKS1), suggesting inhibition of major wall polymers. The fatty acid synthase complex (FAS1/FAS2) bridged sterol and wall clusters, implying systemic disruption of both membrane and wall integrity through impaired lipid precursor supply. This tri-modular interactome indicates that clove oil constituents collectively exert a synergistic, network-level antifungal effect by destabilizing fungal structural and metabolic pathways (Fig. 7).

**Fig. 8:**
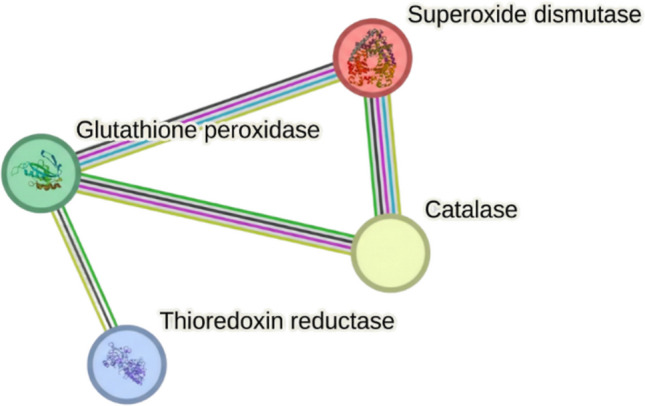
PPI network of the oxidative stress response system in *A. flavus*, showing central antioxidant nodes (SOD, CAT, GPx, TrxR) and the predicted cascading failure induced by selenium nanoparticle (SeNP)-mediated ROS overload

The SeNP-modulated PPI network presented a tightly integrated oxidative stress defense system. Central nodes were defined by SOD and CAT, which detoxify superoxide and hydrogen peroxide, respectively, and were directly connected to glutathione-dependent GPx and thioredoxin-linked TrxR. This architecture revealed a highly interdependent antioxidant network. SeNP-induced ROS generation is predicted to overwhelm SOD capacity, leading to excess superoxide, which elevates hydrogen peroxide flux beyond catalase detoxification limits. Simultaneously, depletion of reduced glutathione and inhibition of peroxidases compromise detoxification of peroxides, while TrxR inhibition collapses thioredoxin recycling and redox signaling. The cumulative effect is a cascading failure of the antioxidant system, resulting in lipid peroxidation, protein carbonylation, and DNA fragmentation, culminating in cell death (Fig. 8).

### Molecular docking analysis of phytochemicals and selenium nanoparticles with *A. flavus *proteins

**Fig. 9:**
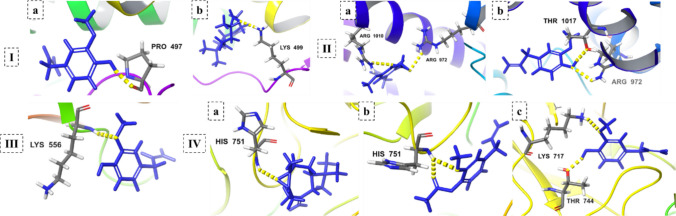
Molecular docking of clove oil constituents against key fungal biosynthetic enzymes. Binding interactions of **I** sterol 14-α demethylase CYP51A with (a) eugenol and (b) caryophyllene oxide; **II** chitin synthase with (a) eugenyl acetate and (b) eugenol; **III** HMG-CoA reductase with eugenol; and **IV** fatty acid synthase β (B8NL80) with (a) caryophyllene oxide, (b) eugenyl acetate, and (c) eugenol

**Fig. 10:**
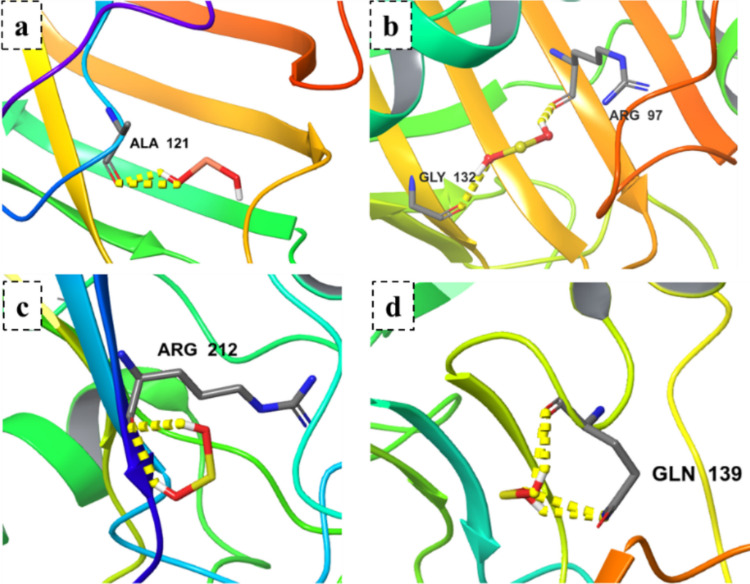
Docking analysis of selenium nanoparticles with fungal oxidative stress enzymes. Interaction profiles of SeNPs against **a** superoxide dismutase, **b** catalase, **c** glutathione peroxidase, and **d** thioredoxin reductase

The docking analysis revealed distinct interaction profiles for clove oil constituents and SeNPs across fungal targets (Figs. 9 and 10). Eugenol showed the strongest binding affinity toward sterol 14-α demethylase (CYP51A, − 5.1 kcal/mol), stabilized by hydrogen bonding with PRO497, suggesting direct interference with ergosterol biosynthesis. Caryophyllene oxide also bound CYP51A (− 4.5 kcal/mol), though with slightly weaker affinity, while eugenyl acetate exhibited moderate interactions with chitin synthase (− 3.6 kcal/mol) through dual hydrogen bonds with ARG972 and ARG1010. Notably, eugenol displayed additional stable docking to HMG-CoA reductase (− 4.3 kcal/mol) via LYS556, indicating disruption of terpenoid backbone biosynthesis. In fatty acid synthase β, caryophyllene oxide (− 4.9 kcal/mol) and eugenyl acetate (− 4.6 kcal/mol) showed significant affinity, interacting with HIS751, while eugenol also engaged multiple residues including LYS717 and THR744.

SeNPs, in contrast, exhibited modest binding scores but targeted the oxidative stress defense system. Catalase (− 3.0 kcal/mol) formed hydrogen bonds with ARG97 and GLY132, while thioredoxin reductase (− 2.8 kcal/mol) interacted with GLN139. Superoxide dismutase (− 2.5 kcal/mol) and glutathione peroxidase (− 2.6 kcal/mol) displayed weaker yet consistent binding. These interactions support SeNP-mediated disruption of antioxidant enzymes, aligning with ROS-induced cellular damage.

## Discussion

Microbial biodeterioration is a critical challenge in the preservation of silver gelatin photographs, whose protein and cellulose-based substrates are highly vulnerable to fungal colonization. Fungi not only degrade the emulsion layer but also cause discoloration, staining, and irreversible loss of image integrity. Environmental instability, particularly high relative humidity and temperature fluctuations, remains the main drivers of microbial proliferation and enzymatic activity in archival collections (Osman et al. 2022; Purkrtova et al. 2022; Gadd et al. 2024). Among the biodeteriogens, *Aspergillus* and *Penicillium* are most frequently encountered across heritage contexts, reflecting their metabolic versatility and ability to thrive on organic cultural materials (Sequeira et al. 2024).

The consistent dominance of *A. flavus* in this study underscores its role as the primary biodeteriogen of gelatin-based photographs, in agreement with reports from both Egyptian and European archives (Osman et al. 2022; Petraretti et al. 2024). Secondary colonizers, such as *A. sojae* and *P. oxalicum*, likely contribute to deterioration through the secretion of hydrolytic enzymes and organic acids, while less abundant fungi, including *A. niger*, *P. chrysogenum*, and *E. nigrum*, mainly affect surface appearance through pigmentation (Purkrtova et al. 2022; Petraretti et al. 2024). Collectively, these findings highlight the need for integrated conservation strategies that combine environmental control with sustainable antifungal interventions.

Molecular identification using ITS sequencing confirmed *A. flavus* as the principal deteriorative fungus in this study. Accurate species-level identification is essential for guiding conservation protocols, and the obtained results align with earlier reports documenting *A. flavus* as a dominant contaminant in archival materials (Abdel-Maksoud et al. 2022; Agrawal et al. 2023).

Evaluation of antifungal treatments demonstrated the superior efficacy of clove essential oil and selenium nanoparticles (SeNPs). Both agents inhibited fungal growth and sporulation, thereby reducing the likelihood of reinfestation. The activity of clove oil can be attributed primarily to eugenol, supported by eugenyl acetate and β-caryophyllene, which collectively disrupt membrane integrity, inhibit ergosterol biosynthesis, and impair lipid metabolism (Tian et al. 2022; Allizond et al. 2023; Liñán-Atero et al. 2024). GC–MS profiling confirmed that these bioactive compounds were present in high abundance, consistent with compositional surveys of clove oils of different origins (Zouirech et al. 2022). The observed reduction in sporulation further strengthens the use of clove oil as an eco-friendly alternative to synthetic fungicides in preventive conservation.

SeNPs exhibited stronger inhibition at lower concentrations, consistent with their unique oxidative mechanism. By catalyzing the generation of ROS, SeNPs overload fungal antioxidant systems, impairing SOD, CAT, and glutathione peroxidase activity, which ultimately leads to oxidative stress, lipid peroxidation, and fungal cell death (Shakibaie et al. 2015; Nowruzi et al. 2023; Saied et al. 2023; Zambonino et al. 2023). This catalytic, non-specific mechanism differs from the pathway-specific inhibition exerted by clove oil constituents. While their complementary nature suggests potential for future combined application, this study evaluated them separately, and experimental validation of compatibility is required before recommending simultaneous use.

Computational and network pharmacology analyses reinforced these experimental findings. Clove phytochemicals were predicted to target multiple biosynthetic modules, particularly sterol metabolism, cell-wall formation, and lipid synthesis, providing a multi-target antifungal strategy that reduces resistance risk (Didehdar et al. 2022; Prajapati et al. 2023). Sesquiterpenes such as caryophyllene oxide further destabilize fungal morphogenesis, while eugenyl acetate supports eugenol’s sustained activity (Silva et al. 2024). In contrast, SeNPs were shown to collapse redox balance by overwhelming ROS detoxification networks, in line with experimental observations of glutathione depletion and antioxidant enzyme inhibition (Mikhailova 2023; Serov et al. 2023; Shahbaz et al. 2023).

Docking studies contextualized these findings within fungal interactomes, demonstrating that clove constituents concentrate inhibitory pressure on sterol and lipid biosynthesis hubs, while SeNPs exert system-level redox disruption. The distinct nature of these mechanisms, specifically biosynthetic pathway inhibition by clove oil versus oxidative stress induction by SeNPs, suggests a theoretical basis for their combined use. However, since this study focused on their independent evaluation, future research must experimentally validate their compatibility and potential synergy (e.g., via Fractional Inhibitory Concentration Index (FICI) determination) before a combined protocol can be recommended. Recent studies confirm that essential oils and nanomaterials can enhance antifungal efficacy (Hassan et al. 2021; Karthik et al. 2024; Dabare et al. 2025).

This study establishes *A. flavus* as the principal fungal threat to silver gelatin photographs and validates clove essential oil and SeNPs as promising, sustainable antifungal treatments. Their mechanisms, biosynthetic pathway inhibition and oxidative stress induction, offer a promising approach for safeguarding photographic heritage, aligning with current conservation strategies that prioritize eco-friendly, high-efficacy interventions.

Although the present study provides an extensive analysis of clove essential oil and selenium nanoparticles as independent antifungal treatments, a limitation of this work is the absence of experimental data regarding their simultaneous application. However, the complementary nature of their mechanisms suggests strong potential for synergy; clove oil targets structural biosynthetic pathways (including ergosterol and cell wall synthesis), while SeNPs induce oxidative stress and disrupt redox balance. A combined treatment may therefore provide enhanced protection by weakening fungal biosynthetic systems while simultaneously overwhelming antioxidant defenses. Future investigations will specifically address this gap by employing checkerboard titration assays to determine the FICI. This quantitative approach aims to identify optimized combinations that allow for the use of lower concentrations of each agent, thereby reducing the risk of substrate alteration while maintaining high antifungal efficacy. These combinations should be validated on both laboratory-prepared and historical photographic samples, coupled with advanced imaging and chemical analyses to assess any potential impact on the gelatin binder and image stability. This would provide a comprehensive understanding of the feasibility of integrated eco-friendly antifungal strategies for the long-term preservation of photographic heritage.

From a practical conservation perspective, surface-spraying of essential oils and nanoparticle-based coatings is an increasingly explored eco-friendly strategy (Menicucci et al. 2023; Pinna 2025). While improper application (e.g., excessive volume) poses a risk of gelatin swelling (Brunelli et al. 2021; Pinna 2025), our protocol mitigated this by utilizing controlled low-volume sprays and non-reactive emulsifiers (Tween 80). This approach preserved the physical integrity of the photographic substrate. Nevertheless, we recommend pre-testing on non-heritage samples before application to original artifacts to ensure specific material compatibility.

## Conclusion

This study confirmed *A. flavus* as the primary biodeteriogen of silver gelatin photographs and demonstrated that both clove essential oil and SeNPs are highly effective antifungal treatments. Clove oil exhibited strong fungistatic and fungicidal activity due to its high content of eugenol and related sesquiterpenes, which impair fungal plasma membranes, ergosterol biosynthesis, and sporulation. In contrast, SeNPs showed superior efficacy at lower concentrations, acting through oxidative stress induction and disruption of fungal antioxidant defenses. The novelty of this work lies in the first comparative evaluation of clove oil and SeNPs applied directly to photographic materials, combined with mechanistic insights from computational analysis. While a limitation of this study is the absence of experimental data on their combined application, the results establish a necessary baseline for future synergistic protocols. Ultimately, this study highlights both treatments as promising eco-friendly alternatives to synthetic fungicides, offering safe and effective strategies for protecting silver gelatin photographic heritage from biodeterioration.

## Supplementary information

Below is the link to the electronic supplementary material.

ESM 1 (DOCX 6.22 MB)

## Data Availability

All data generated or analysed during this study are included in this published article and supplementary file. The ITS gene sequence generated in this study has been deposited in the GenBank database under accession number MZ945517.
