# Supplementary material for: Clove oil and selenium nanoparticles as eco-friendly antifungal agents for photographic preservation
Source: Appl Microbiol Biotechnol. 2026 Feb 21;110(1):72. doi: 10.1007/s00253-026-13730-3 (PMC12924790; doi:10.1007/s00253-026-13730-3)
Supplement: Supplementary file 1 — (DOCX 6.22 MB) [file 253_2026_13730_MOESM1_ESM.docx]

**Supplemental Material**

**Applied Microbiology and Biotechnology**

**Clove oil and selenium nanoparticles as eco-friendly antifungal agents for photographic preservation**

Heba El-Sayed*****^1^, Mohamed E. Osman^1^, Nesma Ali, Eslam T. Mohamed^1^

^1^Botany and Microbiology Department, Faculty of Science, Helwan University, Helwan, 11795, Egypt. [HebaElsayed@science.helwan.edu.eg](mailto:HebaElsayed@science.helwan.edu.eg), [mesosman@hotmail.com](mailto:mesosman@hotmail.com), [EslamElsaaid@science.helwan.edu.eg](mailto:EslamElsaaid@science.helwan.edu.eg)

### ^2^ Ministry of Tourism and Antiquities, Egypt. [t_nesma_r@hotmail.com](mailto:t_nesma_r@hotmail.com)

*****Corresponding authors: Heba El-Sayed, Email: [HebaElsayed@science.helwan.edu.eg](mailto:HebaElsayed@science.helwan.edu.eg); [drhhebaelsayed39@gmail.com](mailto:drhhebaelsayed39@gmail.com)

**
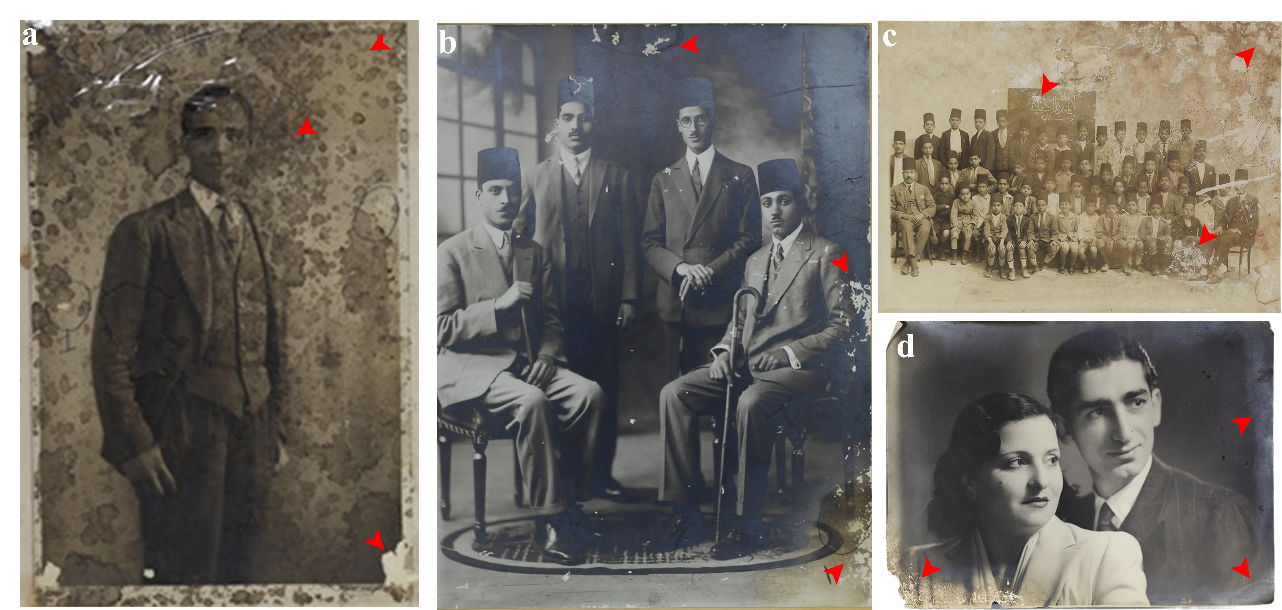
**

**Fig. S1** Historical silver gelatin photographs showing biodeterioration signs: (a) gelatin layer detachment and edge cracking; (b) brown staining and localized fading; (c) surface abrasion and pigment loss; (d) severe emulsion deterioration and discoloration. Arrows indicate areas of visible fungal damage.


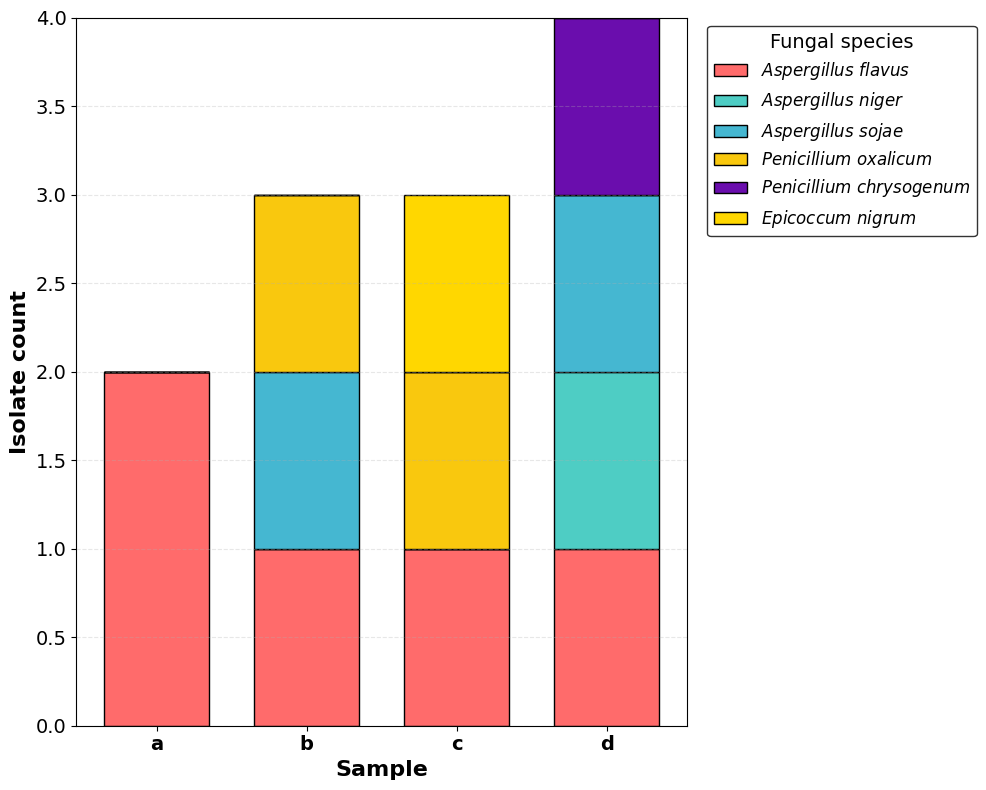


**Fig. S2** Isolate count of fungal species recovered from four historical silver gelatin photographs. The samples are designated as: (a) Full-length portrait of a standing man; (b) Group portrait of three men in formal attire; (c) Large group photograph mounted on cardstock; (d) Portrait of a couple; The stacked bar chart displays the frequency of fungal taxa isolated from each photograph.

**Fig. S3** Phylogenetic tree based on ITS sequences of rDNA of the fungal sample isolated from the biodegraded black and white gelatin photos (*A. flavus* AUMC15234 with GenBank accession no. MZ945517, arrowed) aligned with closely related strains accessed from the GenBank (GB). It showed 100% identity and 100% coverage with several strains of *Aspergillus flavus* including the type strain ATCC 16833 (GB no.: NR_111041). *Aspergillus candidus* ATCC 1002 was used as the outgroup. (*A.*= *Aspergillus*).
